# Cytotoxic effect of HIV-1 gp120 on primary cultured human retinal capillary endothelial cells

**Published:** 2011-12-28

**Authors:** Haotian Lin, Weirong Chen, Lixia Luo, Changrui Wu, Qilin Wang, Yizhi Liu

**Affiliations:** State Key Laboratory of Ophthalmology, Zhongshan Ophthalmic Center, Sun Yat-sen University, Guangzhou, China

## Abstract

**Purpose:**

This research was conducted to make a primary culture of human retinal capillary endothelial cells (HRCEC) and to study the cytotoxic effect of human immunodeficiency virus-1 (envelope) glycoprotein 120 (HIV-1 gp120) on cultured HRCEC.

**Methods:**

HRCEC were isolated and primarily cultured as dissociated single cell cultures. Immunohistochemistry and immunofluorescence were used to identify specific markers of HRCEC and to reveal HIV-1 gp120 related receptors (cluster of differentiation 4 [CD4], C-X-C chemokine receptor type 4 [CXCR4], and C-C chemokine receptor type 5 [CCR5]). The 3-[4,5-dimethylthiazol-2-yl]-2,5-diphenyl tetrazolium bromide (MTT) assay was used to demonstrate the effect of HIV-1 gp120 on cell viability at seven different concentrations (0.01–0.15 mg/l) for 24 h or at a fixed concentration of 0.08 mg/l for varying time intervals (4–72 h). After 0.08, 0.1, 0.12, and 0.15 mg/l HIV-1 gp120 were applied to HRCEC for 24 h, cell apoptotic rates and the mitochondrial membrane potential were measured with flow cytometry; pro-caspase-9 and cleaved caspase-9 were evaluated with immunoblotting. Under each research condition, 0.15 mg/l of HIV-1 gp120 mutated proteins (423 I/P) were used as controls.

**Results:**

Primary cultures of pure HRCEC were established, and the cells were characterized with their specific markers. HIV-1 gp120 receptors CXCR4 and CCR5 were found on the cell surface of HRCEC; however, CD4 was negative. Treatment of HRCEC with HIV-1 gp120 at concentrations <0.08 mg/l did not influence cell viability. However, a concentration- and time-dependent increase of HIV-1 gp120-induced cell inhibition was demonstrated with MTT, when the concentration of HIV-1 gp120 was more than 0.08 mg/l (r=-0.763, p<0.01). With increasing concentrations of HIV-1 gp120, the numbers of apoptotic cells and expression of cleaved caspase-9 protein increased, but Rho123 staining mitochondrial membrane potential decreased.

**Conclusions:**

HIV-1 gp120 assistant receptors CXCR4 and CCR5 are expressed on the cell surface of HRCEC, and HIV-1 gp120 can inhibit cell viability and induce apoptosis of HRCEC. The mitochondrial pathway is probably involved in HIV-1 gp120-induced apoptosis of HRCEC, but the specific mechanisms remain to be uncovered.

## Introduction

Along with the increasing numbers of patients with human immunodeficiency virus type 1 (HIV-1), HIV-1-related eye diseases have become a great challenge for ophthalmologists all over the world. Severe retinopathy and uveitis [1] are the main untreatable causes of ablepsia in these patients, since the pathogenesis has remained unclear until now. However, researchers have believed that disruption of the structure and function of the blood-retina barrier (BRB) is the primary cause [2-5]. The BRB consists of the outer barrier (retina pigment epithelial cells and their tight junctions) and the inner barrier (retina capillary endothelial cells and their tight junctions), both of which are important in maintaining the integrity and normal function of the BRB. Our previous research focused on the destruction of the outer barrier by HIV-1 proteins [6,7], but further research about the inner barrier was delayed until we could successfully establish primary cultures of inner blood-retina barrier cells [8]. Whether the destruction of the inner barrier is more important than that of the outer barrier to HIV-1 retinopathy has not been proved.

It is well known that HIV-1 infections involve binding of the viral external envelope glycoprotein (gp120) to cell-surface cluster of differentiation 4 (CD4) molecules, followed by interactions with coreceptors of C-X-C chemokine receptor type 4 (CXCR4) and C-C chemokine receptor type 5 (CCR5; T cells and macrophage cell-surface binding of the natural chemokine receptors), which results in the fusion of the viral and cellular membranes [9]. In addition, previous studies demonstrated that dissociated HIV-1 gp120 in blood is toxic to cells through the HIV-1 related coreceptor (CXCR4 and CCR5), inducing oxidative stress, inflammatory cytokines, apoptosis, and tight junction injury [10,11]. Our previous studies revealed that HIV-1 gp120 could induce oxidative stress in human retina pigment epithelial cells [6], but the influence of HIV-1 gp120 on human retinal capillary endothelial cells (HRCEC) and its mechanism remained unknown. To clarify this question in the current research, we first established primary cultures of HRCEC identified by specific markers (von Willebrand factor and zonula occludens-1). At the same time, the expression of HIV-1 related receptors (CD4, CXCR4, and CCR5) was investigated, and further we exposed primary cultured HRCEC to HIV-1 gp120. Thereafter, the cytotoxic effect of HIV-1 gp120 on cultured HRCEC was investigated to explore the possible molecular mechanism.

## Methods

### Reagents

Gp120, obtained from the National Institutes of Health AIDS Reagent Program (Rockville, MD), was a generous gift from Prof. Hui Zhang of Thomas Jefferson University (Philadelphia, PA). Dulbecco’s modified Eagle’s medium/High Glucose (DMEM), fetal bovine serum (FBS), penicillin, and streptomycin were purchased from Hyclone (Logan, UT). Rat antihuman CD4, CXCR4, and CCR5 monoclonal antibodies were purchased from R&D Systems (Shanghai, China). Rabbit antibodies against (cleaved) caspase-9 antibody (No. 9501-Asp330, No. 9502) were obtained from Cell Signaling Technology (Danvers, MA). Except for those indicated in the text, all other chemicals and reagents were obtained from Invitrogen/Gibco (Grand Island, NY).

### Tissue source, primary culture, and identification of HRCEC

This study was performed in accordance with the ethical standards laid down in the 1964 Declaration of Helsinki for Research Involving Human Tissue. After receiving consent for research use and Sun Yat-sen University ethics committee approval, six postmortem eyes (donors age 24–32) were obtained from the Eye Bank of Zhongshan Ophthalmic Center (Guangzhou, China) 8–24 h after death. Only eyes free of ocular pathology were used to establish primary cultures. The research was conducted according to the tenets of the Declaration of Helsinki.

The methods for isolating, culture, and identifying HRCEC were described in our recent published paper [8], which was focusing on primary culture of human retina cells. To investigate HIV-1 related receptors (CD4, CXCR4, and CCR5) in HRCEC, antibodies against CD4, CXCR4, and CCR5 were used and analyzed with immunofluorescent staining and immunocytochemistry microscopy.

### Preparation and treatment of gp120 protein

A solution of gp120 with a concentration of 2 g/l was made by adding 100 µg freeze-dried powder of gp120 in 50 µl PBS. Then the prepared solution was stored at −20 °C, and was diluted with serum-free culture fluid when used. HRCEC were cultured in the plastic plates (Sigma, St. Louis, MO) until subconfluence and treated with gp120 at concentrations [12] of 0.01, 0.02, 0.04, 0.08, 0.1, 0.12, and 0.15 mg/l for 24 h, or with 0.08 mg/l of HIV-1 gp120 solution culture for different periods of 4, 8, 12, 24, 48, and 72 h. In the control group, HRCEC were treated with 0.15 mg/l of HIV-1 gp120 mutated proteins (423 I/P), which did not bind to CD4 or CXCR4/CCR5 as a result of the disruption of the HIV-1 gp120 bridging sheet [13], and the other conditions were the same as each research group. Each experiment was repeated at least three times.

### Cell viability assay

Cells were grown in 96-well plates at a density of 1×10^4^ cells/well. After the indicated treatments, 3-[4,5-dimethylthiazol-2-yl]-2,5-diphenyl tetrazolium bromide (MTT) was added to a final concentration of 5 g/l to each well for 4 h, after which the culture medium was removed and 150 μl of dimethyl sulfoxide (DMSO) was added to each well. Absorbance at 490 nm was measured using a multifunctional microplate reader (POLARstar OPTIMA, BMG Labtechnologies, Ortenberg, Germany).

### Evaluation of apoptosis

Apoptotic rates were analyzed with flow cytometry (FACS) using the Annexin V-fluorescein isothiocyanate (FITC)/propidium iodide (PI) kit (Sigma, St. Louis, MO), in which annexin V binds to the exposed phosphatidylserine on the plasma membrane of apoptotic cells. Staining was performed according to the manufacturer’s instructions provided by Sigma Company (Sigma), and FACS was conducted on a FACS Caliber (Becton Dickinson, Mountain View, CA). The percentage of the early apoptosis was calculated from the proportion of cells that was annexin V-positive but PI-negative, while the percentage of the late apoptosis plus necrosis was calculated from the proportion of cells that was annexin V- and PI-positive.

### Measurement of mitochondrial membrane potential

Loss of the mitochondrial membrane potential (ΔΨm) was assessed with flow cytometry using the fluorescent indicator Rhodamine123 (Rho123; Sigma), as previously described [14]. Briefly, Rho123 working solution was added to the culture to a final concentration of 2 g/l, and then the culture was incubated in the dark for 30 min at 37 °C. Cells were then washed with PBS and evaluated immediately for Rho123 fluorescence using a FACS Caliber, at an excitation wavelength of 488 nm and an emission wavelength of 525 nm.

### Western blot analysis

Cells were lysed with 20 ml of ice-cold lysis buffer (50 mM HEPES, 5 mM EDTA [EDTA], 100 mM NaCl, 1% Triton X-100 pH 4) in the presence of a protease inhibitor cocktail (Roche, Berlin, Germany). Protein concentrations were determined with the bicinchoninic acid (BCA) protein assay kit (Pierce, Rockford, IL). Protein samples (20 μg) were resolved on 10% sodium dodecyl sulfate PAGE (SDS–PAGE) gels and transferred onto a polyvinylidene difluoride (PVDF) membrane (Millipore, Bedford, MA) using a semi-dry system (Bio-Rad, Hercules, CA). The membranes were incubated with specific antibodies against pro-caspase-9 (No. 9502), cleaved caspase-9 (Asp330 No. 9501; 1:100) and β-actin (1:500). β-actin was used as a loading control in experiments with cellular proteins. Chemiluminescence was visualized by exposure to X-ray films. The optical densities of the bands were scanned and quantified with Gel Doc 2000 (Bio-Rad). Data were normalized to those of the corresponding β-actin levels.

### Statistical analysis

Data are expressed as the mean±SD. Differences between groups were assessed with one-way ANOVA, and the correlation of bivariate between the concentration of gp120 and its cytotoxic effect were analyzed with a statistical software program (SPSS 17.0; SPSS, Chicago, IL). p<0.05 was considered indicative of statistical significance.

## Results

### Primary culture and identification of HRCEC

HRCEC were cultured, maintained, and passaged using specially designed media and following the procedures optimized by our research group as described previously [8]. The purity of HRCEC was analyzed with immunochemistry staining and immunofluorescence microscopy and confirmed with FACS with antibodies against its specific markers. As indicated in our previous research [8], primary HRCEC expressed von Willebrand factor and zonula occludens-1.

### Investigation of HIV-1 gp120 related receptors in HRCEC

**Figure 1:**
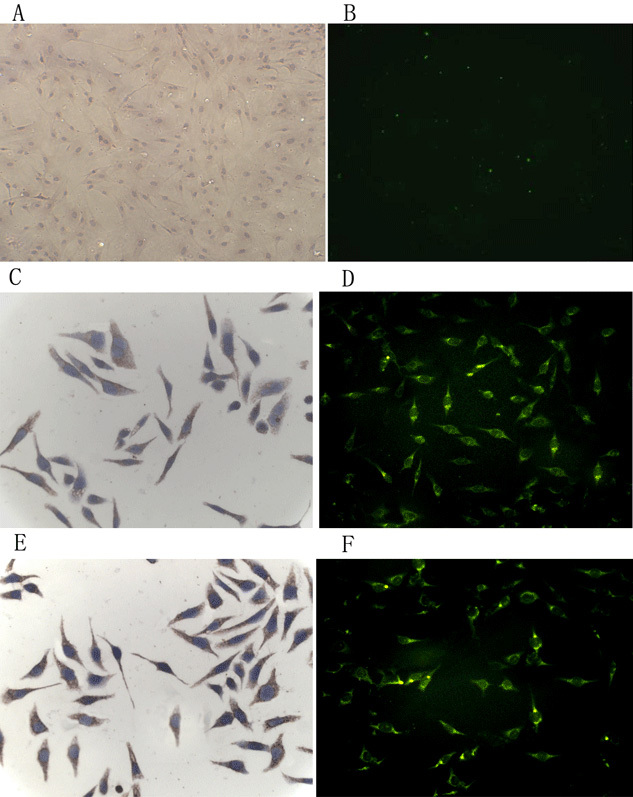
Expression of CD4, CXCR4, and CCR5 in HRCEC. CXCR4 (**C** and **D**) and CCR5 (**E** and **F**) receptors were positive on the cell surface of HRCEC, but the CD4 receptor (**A** and **B**) was negative. **A**, **C**, and **E** were by immunocytochemistry; **B**, **D**, and **F** were by immunofluorescent staining. To focus on specific markers in immunofluorescent staining, 4', 6-diamidino-2-phenylindole (DAPI) nuclear staining on **B**, **D**, and **F** was not displayed. Three independent experiments were performed.

According to the immunocytochemistry and immunofluorescence, CXCR4 and CCR5 receptors were found on the cell surface of HRCEC, while the CD4 receptor was not. Data are shown in Figure 1.

### Effect of HIV-1 gp120 on cell viability of HRCEC

**Figure 2:**
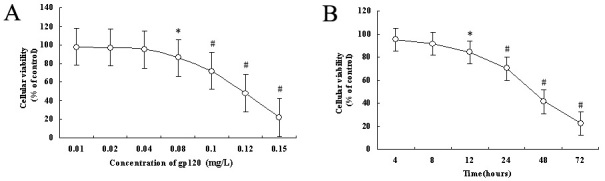
Effects of HIV-1 gp120 on the cell viability of HRCEC. **A**: Dose–response effects of HIV-1 gp120 for 24 h in an MTT assay. **B**: Time course of the effects of HIV-1 gp120 on HRCEC. HRCEC were incubated with 0.08 mg/l of HIV-1 gp120 for the times indicated. Cellular viability=(absorbance rate of study group÷absorbance rate of control group) ×100%.*p<0.05, # p<0.01 compared with control (HIV-1 gp120 mutated proteins, 423 I/P). Each data point represents the mean±SD of three independent experiments.

To determine the cytotoxicity of HIV-1 gp120 on HRCEC, we used the concentration gradient of gp120 and various exposure durations to demonstrate the cytotoxicity with MTT assay. In groups treated with low concentrations (<0.08 mg/l) of gp120 protein, HIV-1 gp120 protein demonstrated no significant effect on the viability of HRCEC. However, in groups with a concentration of gp120 protein exceeding 0.08 mg/L, the HIV-1 gp120 protein appeared to significantly inhibit the cell viability of HRCEC in 24 h, showing a clear dose-dependent manner in Figure 2A (r=-0.763, p<0.001). In groups treated with a fixed concentration of 0.08 mg/l, HIV-1 gp120 could significantly inhibit the viability of HRCEC after 12 h culture, showing a time dependence in Figure 2B (r=-0.833, p<0.001).

### Induction of apoptosis in HRCEC by HIV-1 gp120

**Figure 3:**
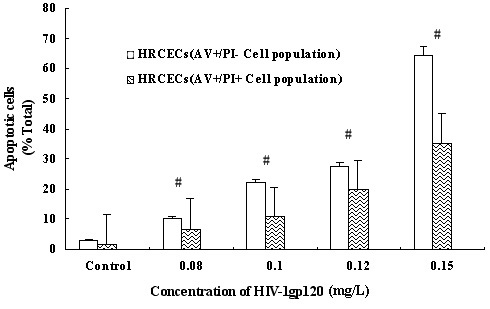
Apoptotic cell death of HRCEC treated with HIV-1 gp120. After being treated with gp120 at concentrations of 0.08, 0.1, 0.12, and 0.15 mg/l for 24 h, apoptotic cells were analyzed using the annexin V-FITC/PI double marked method by FCM. Early apoptotic cells were annexin -positive but PI-negative (left blank plots); late apoptotic ells were annexin V- and PI-positive (right corrugated plots). The ratio of apoptotic cells increased significantly with the increase of protein concentration. #p<0.01 compared with control. Data represent the mean±SD of three independent experiments.

To clarify the mechanism of gp120-induced cytotoxicity on HRCEC, we examined early and late apoptosis in HRCEC using annexin V-FITC and PI double labeling of living cells. Annexin V binds to phosphatidylserine exposed on the cell membrane, one of the earliest indicators of cellular apoptosis. As shown in the plots in Figure 3, FACS analysis revealed that the numbers of early and late apoptotic cells increased with gp120 exposure in a dose-dependent manner. When the concentration of gp120 was >0.08 mg/l, the difference was significant compared with control.

### Involvement of the mitochondrial pathway in HIV-1 gp120-induced apoptosis

**Figure 4:**
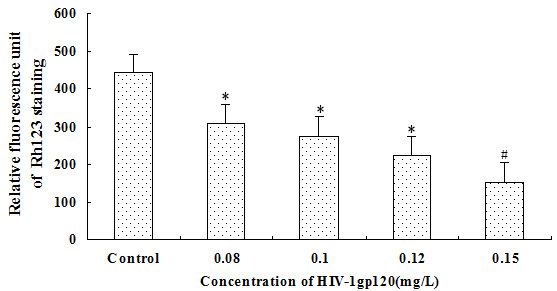
Collapse of mitochondrial membrane potential (ΔΨm) measured with FCM in HRCEC treated with HIV-1 gp120. The fluorescence signal of Rho123 decreased significantly with the increase of the HIV-1 gp120 concentration. *p<0.05, # p<0.01 compared with control. Data represent the mean±SD of three independent experiments.

Mitochondria play a crucial role in regulating cell death, and the loss of ΔΨm is an irreversible event in the mitochondrial apoptotic pathway. To confirm whether gp120 causes the loss of ΔΨm in HRCEC, we evaluated Rho123 fluorescence in the cells, while the depolarization of ΔΨm was assessed by the reduced Rho123 accumulation in the mitochondria. We employed this indicator to estimate the effect of gp120 on the ΔΨm. As shown in Figure 4, treatment of cells for 24 h with 0.08, 0.1, 0.12, and 0.15 mg/l of gp120 led to significantly decreasing Rho123 fluorescence in a dose-dependent manner (r=-0.842, p<0.001).

**Figure 5:**
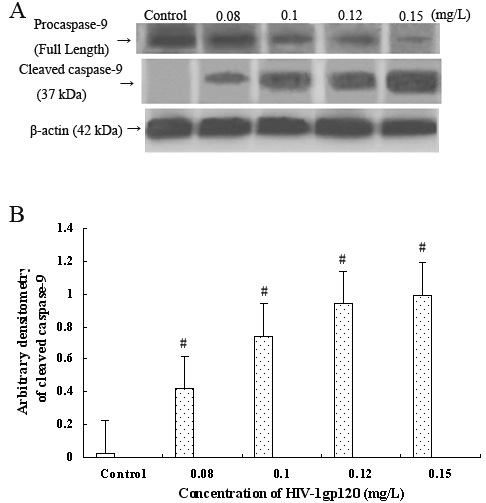
Expression of caspase-9 in HRCEC with or without HIV-1 gp120 treatment. **A**: western blot showed the expression of pro-caspase-9 (full length, using antibody No. 9502) and cleaved caspase-9 (37 kDa, using antibody No. 9501-Asp330) in HRCEC without or with gp120 treatment. The activation of caspase-9 is reflected by the appearance of the bands representing the 37-kDa cleavage product. **B**: The densities of the bands were quantified with Bio-Rad Gel Doc 2000. Arbitrary densitometry of cleaved caspase-9 show that its expression increases significantly with the increase of HIV-1 gp120 protein concentration. #p<0.01 compared with control. Data represent the mean±SD of three independent experiments.

However, caspase-9 activation also occurs mainly via the mitochondrial pathway, so we tested the effect of gp120 on the activation of caspase-9 at the same time. As shown in Figure 5, treatment with gp120 caused significantly increasing cleavage of pro-caspase-9 in a dose-dependent manner (r=0.792, p<0.001). These two results together indicate that HIV-1 gp120 induces apoptosis of HRCEC most likely via the mitochondrial pathway, though the specific mechanism remains to be studied.

## Discussion

HIV-1-associated retinopathy (disruption of the blood-retina barrier) is a great threat to human visual function and quality of life [15]. The most likely effect of blood-retina barrier disruption on infections is increased access to ocular tissues and perhaps increased access of immune cells to the eye [3,16]. However, the specific mechanisms of the impact of HIV-1 on the human blood-retinal barrier are not yet known [17].

Gp120, a highly glycosylated hydrophilic envelope protein of HIV-1, located on the virus particles and infected cell surface, or just in free form, has the ability to target the cell surface of CD4 and CXCR4/CCR5 receptors [18-20]. Gp120 involves many types of cells and may cause injury and even apoptosis of different cells, even without the CD4 receptor [21]. Ullrich et al. [22] reported that binding of gp120/160 with CXCR4 induces caspase-dependent apoptosis of human umbilical endothelial cells, which are not CD4^+^ cells but uninfected CXCR4-positive bystander cells. Researchers have also demonstrated that the viral envelope glycoprotein gp120 plays a key role in the process of HIV-1 violation of blood-brain barrier [23-25]. In our study, we found that the CD4 receptor was not expressed on HRCEC, but CXCR4/CCR5 receptors were indeed expressed on the cell surface of HRCEC. Although the source of the cells in Ullrich’s study (human umbilical endothelial cells) was different from that in our study [22], our results (HRCEC, newly proved uninfected CXCR4/CCR5-positive bystander cells) confirm Ullrich’s finding, and suggest the gp120-induced damage to HRCEC is also probable mediated by the CXCR4/CCR5 receptor.

To appropriately demonstrate the toxic effects of HIV-1 gp120 protein on HRCEC, we have to find a suitable concentration. From the present studies about gp120 cellular toxicity, the concentration range varied widely, from 1 pM to 10 uM (0.12 ng/ml–120 mg/l range, 1 nM=0.12 mg/l). However, the clinical studies reported by Oh et al. [26] that the concentration of gp120 in plasma of patients with acquired immune deficiency syndrome was 0.1–0.8 nM (0.012–0.096 mg/l). Therefore, we chose to use the concentration of gp120 from 0.01 to 0.15 mg/l, which covered the concentration range in the actual situation of patients with acquired immune deficiency syndrome. When the protein levels are >0.08 mg/l, HIV-1 gp120 induced significant inhibition of the cell viability of HRCEC in a dose- and time-dependent manner. With the increasing concentration, the rate of HRCEC apoptosis statistically significantly increased compared to the control group.

As we know, mitochondria are the core of the intrinsic apoptosis pathway, storing many cysteinyl aspartate specific proteinase–induced activation and chromosome breakage proteins [27,28]. The role of mitochondria in apoptosis includes the release of caspase-activating factors such as cytochrome C, the loss of electron transfer function and reduction in energy generation, the disappearance of ΔΨm as well as the related function of the Bcl-2 family of proteins to promote and inhibit apoptosis. The mitochondrial participation in apoptosis is connected with the collapse of the ΔΨm, which is considered the “point of no return” in the death cascade [29]. Our results demonstrated that HIV-1 gp120 enhanced the collapse of ΔΨm and caspase-9 activation, which may lead to a significant increase in mitochondrial breakdown. In all, these results indicated that the mitochondrial pathway is probably involved in HIV-1 gp120-induced apoptosis of HRCEC, but the specific mechanism remains to be studied.

Taken together, the present findings show that HIV-1 gp120 assistance receptors of CXCR4 and CCR5 are expressed on the cell surface of HRCEC (new defined uninfected CXCR4/CCR5-positive bystander cells), and HIV-1 gp120 is cytotoxic to cultured HRCEC in a dose- and time-dependent manner. The subsequent analysis of FACS with annexin V-FITC and PI labeling shows that HRCEC death induced by different concentrations of HIV-1 gp120 proceeds through the apoptotic pathway. The mechanism involves the loss of ΔΨm and activation of pro-caspase-9, indicating that the mitochondrial pathway is probably associated with gp120-induced apoptosis. Further investigation in CXCR4/CCR5 interaction with the HIV-1 envelope and the mitochondria pathway of HRCEC apoptosis may ultimately prove the specific mechanisms of HIV-1 related retinopathy.
